# Clinical Evaluation of Tuberculosis Viability Microscopy for Assessing Treatment Response

**DOI:** 10.1093/cid/ciu1153

**Published:** 2014-12-23

**Authors:** Sumona Datta, Jonathan M. Sherman, Marjory A. Bravard, Teresa Valencia, Robert H. Gilman, Carlton A. Evans

**Affiliations:** 1 Innovation for Health and Development (IFHAD), Laboratory for Research and Development, Universidad Peruana Cayetano Heredia, Lima, Peru; 2 Infectious Diseases and Immunity and Wellcome Trust Centre for global Health Research, Imperial College London, United Kingdom; 3 Innovacion por la Salud y el Desarollo (IPSYD), Asociación Benéfica Prisma, Lima, Peru; 4 Department of International Health, Johns Hopkins Bloomberg School of Public Health, Baltimore, Maryland

**Keywords:** fluorescein diacetate, viability stain, vital stain tuberculosis, early bactericidal activity, multidrug-resistant tuberculosis

## Abstract

***Background.*** It is difficult to determine whether early tuberculosis treatment is effective in reducing the infectiousness of patients' sputum, because culture takes weeks and conventional acid-fast sputum microscopy and molecular tests cannot differentiate live from dead tuberculosis.

***Methods.*** To assess treatment response, sputum samples (n = 124) from unselected patients (n = 35) with sputum microscopy–positive tuberculosis were tested pretreatment and after 3, 6, and 9 days of empiric first-line therapy. Tuberculosis quantitative viability microscopy with fluorescein diacetate, quantitative culture, and acid-fast auramine microscopy were all performed in triplicate.

***Results.*** Tuberculosis quantitative viability microscopy predicted quantitative culture results such that 76% of results agreed within ±1 logarithm (*r*_S_ = 0.85; *P* < .0001). In 31 patients with non-multidrug-resistant (MDR) tuberculosis, viability and quantitative culture results approximately halved (both 0.27 log reduction, *P* < .001) daily. For patients with non-MDR tuberculosis and available data, by treatment day 9 there was a >10-fold reduction in viability in 100% (24/24) of cases and quantitative culture in 95% (19/20) of cases. Four other patients subsequently found to have MDR tuberculosis had no significant changes in viability (*P* = .4) or quantitative culture (*P* = .6) results during early treatment. The change in viability and quantitative culture results during early treatment differed significantly between patients with non-MDR tuberculosis and those with MDR tuberculosis (both *P* < .001). Acid-fast microscopy results changed little during early treatment, and this change was similar for non-MDR tuberculosis vs MDR tuberculosis (*P* = .6).

***Conclusions.*** Tuberculosis quantitative viability microscopy is a simple test that within 1 hour predicted quantitative culture results that became available weeks later, rapidly indicating whether patients were responding to tuberculosis therapy.

## Abstract (precis)

Tuberculosis viability microscopy predicted, within 1 hour, quantitative culture results that became available weeks later. Viability microscopy provides promising results for informing decisions concerning drug susceptibility testing, treatment changes, and infection control measures in resource-constrained settings where most tuberculosis occurs.

**(See the Editorial Commentary by Lawn and Nicol on pages 1196–8.)**

Concentrations of viable *Mycobacterium tuberculosis* in patients' sputum are demonstrated by quantitative culture to decline rapidly during the first days of adequate tuberculosis treatment [1]. Quantifying this early treatment response identifies patients whose treatment is inadequate [1–3], potentially allowing drug susceptibility testing (DST) to be provided sooner to patients most likely to benefit. This is important because, although universal DST is a priority, resources are so limited that globally only 5% of tuberculosis patients are tested [4]. Assessing early treatment response may help detect not only drug resistance, but also other causes of incipient treatment failure including malabsorption and poor adherence, which are difficult to detect.

There is no appropriate-technology test for quantifying viable *M. tuberculosis* in sputum. Quantitative culture is technically demanding, infrequently available, and provides results that are weeks out of date [5]. The GeneXpert MTB/RIF assay [6] and acid-fast microscopy using the Ziehl-Neelsen or auramine stains do not differentiate viable from nonviable *M. tuberculosis*, so they cannot assess early treatment response [7]. Molecular tests quantifying ribonucleic acid in viable *M. tuberculosis* are not feasible in resource-constrained settings [8]. Consequently the detection of failing tuberculosis treatment is often delayed, risking morbidity, mortality, and contagion [9].

Fluorescein diacetate is a viability stain that fluoresces only after hydrolysis by nonspecific esterases in the cytoplasm of viable, metabolically active bacteria [10]. Fluorescein diacetate microscopy assesses the viability of microorganisms, including the mycobacteria that cause leprosy and tuberculosis [10, 11]. Fluorescein diacetate has recently been used with tuberculosis sputum microscopy to predict culture positivity [12, 13] and with flow cytometry to determine drug susceptibility [14–16].

We hypothesized that sputum tuberculosis quantitative viability microscopy (henceforth termed “viability”) would predict the concentration of culturable *M. tuberculosis*, indicating whether patients are responding to tuberculosis treatment, potentially informing clinical care and infection control decisions. To test these hypotheses, we optimized viability microscopy and compared results with culture and acid-fast microscopy during early treatment. We have presented early findings [17–19] and here report final results.

## MATERIALS AND METHODS

### Ethics

All participants gave informed written consent. The internationally accredited ethics committee of the Universidad Peruana Cayetano Heredia approved the study. All clinically relevant results were provided to participants and their physicians in collaboration with the Ministry of Health. This research had no role in patient care, which was free for all patients with suspected tuberculosis and was not delayed or modified by participation. Patients received empiric first-line tuberculosis therapy with clinic-based direct observation of every dose from the tuberculosis program, according to Peruvian policy, as detailed in Supplementary Table 1 [20, 21].

#### Inclusion Criteria

Inclusion criteria were unselected adults diagnosed with sputum smear microscopy–positive tuberculosis disease in shantytowns in Ventanilla, Peru [22]. Patients also had to be available to collect the first baseline sputum sample prior to commencing treatment, and all sputa were collected over 12 hours to reduce random variation between samples.

### Specimens

Single sputum samples were collected pretreatment (day 0), and on treatment days 3, 6, and 9. DST results became available and could influence treatment only after these samples were collected.

#### Patients

Patients were interviewed at recruitment from 2006 to 2007 (Table 1). Radiography was not a routine part of tuberculosis management and was not studied [20]. We made follow-up visits every 3 years until 2013 to ask whether tuberculosis had recurred, screen ex-patients for respiratory symptoms, and offer symptomatic ex-patients tuberculosis testing with sputum culture. We categorized treatment outcome as “good” (cured or completed treatment without treatment failure as assessed by the tuberculosis program, and with no recurrence) or “adverse” (treatment failure, noncompletion, death during treatment, or recurrence) [23, 24].

**Table 1.** Baseline Patient and Laboratory Data

Characteristic	Day 0	Day 3	Day 6	Day 9
Laboratory results				
Samples collected, No.	35	34	30	25
Viability microscopy positive, % (no./No.)	94% (33/35)	76% (26/34)	53% (16/30)	24% (6/25)
Culture positive, % (no./No.)	100% (33/33)^a,b^	94% (29/31)^a^	81% (21/26)^a^	64% (14/22)^a^
Acid-fast microscopy positive, % (no./No.)	100% (35/35)	97% (33/34)	97% (29/30)	88% (22/25)
Multidrug resistant, % (no./No.)	12% (4/35)			
Isoniazid monoresistant, % (no./No.)	5.60% (2/35)			
Rifampicin monoresistant, % (no./No.)	0.0% (0/35)			
Ethambutol resistant, % (no./No.)^c^	37% (13/35)			
Streptomycin resistant, % (no./No.)^c^	34% (12/35)			
Ciprofloxacin resistant, % (no./No.)^c^	0.0% (0/35)			
Capreomycin resistant, % (no./No.)^c^	2.9% (1/35)			
Patient characteristics				
Age, median, y (IQR)	26 (22–35)			
Sex, % male (no./No.)	57% (20/35)			
Body mass index, mean (SD)	21 (2.7)			
Poverty indicator: median food spending, US$/week (IQR)	6.3 (4.0–8.7)			
BCG, % with scar (no./No.)	94% (30/35)			
Past tuberculosis diagnosis, % (no./No.)	6.30% (2/35)			
HIV, % (no./No.)	0.0% (0/35)			
Characteristics of illness				
Productive cough, % (no./No.)	94% (30/35)			
Fever, % (no./No.)	69% (22/35)			
Night sweats, % (no./No.)	72% (23/35)			
Days with symptoms, median (IQR)	30 (20–60)			

Patient and laboratory data are shown at the time of recruitment (day 0) and on days 3, 6, and 9 of treatment. Denominators vary because of 11% missing samples and 10% contaminated quantitative cultures.

Abbreviations: BCG, bacillus Calmette-Guerin; HIV, human immunodeficiency virus; IQR, interquartile range; no., number of participants with that characteristic; No., number of participants with available data; SD, standard deviation.

^a^ Twelve quantitative culture results were unavailable because of contamination of the cultures.

^b^ All pretreatment (day 0) samples were culture-positive in conventional nonquantitative microscopic-observation drug susceptibility (see “Results” section).

^c^ Drug susceptibility results from tetrazolium microplate assay only.

### Processing

Sputum was collected in patients' homes at room temperature, transported at 4**°**C, and decontaminated within 24 hours by mixing 2 mL of sputum with 2 mL 4% sodium hydroxide, 2.9% sodium citrate, 0.5% *N*-acetyl-*L*-cysteine [25]. Reagents were obtained from Sigma. After 20 minutes, excess phosphate-buffered saline (PBS; pH 6.8) was added and centrifuged (15 minutes at 3000*g*); the pellet was resuspended in 2 mL PBS, immediately smeared onto microscope slides with 10 µL bovine serum albumin for adhesion, and dried at room temperature. Samples then underwent viability microscopy, quantitative culture, and acid-fast microscopy, each in triplicate. This standardized decontamination protocol was used, and the exact smear area was marked on slides with a hydrophobic pen to reduce processing variability between the triplicate smears.

### Viability

One hundred microliters of decontaminated sputum was smeared over 1 cm^2^ area of the microscope slide (20 times greater density than for acid-fast microscopy). Fluorescein diacetate staining [13] used a stock 5 mg in 1 mL diluent (40% acetone in PBS) solution, which was diluted daily to 20 µg/mL in diluent and soaked onto a 1 cm^2^ piece of Whatman grade 3 filter paper that was incubated on the smear at 37°C for 20 minutes. The paper was discarded, the slide allowed to dry in the dark for 10 minutes, and microscopy performed immediately. Concurrent nonviable counterstaining was not used.

### Acid-Fast Staining

Ten microliters of decontaminated sputum was smeared over 2 cm^2^ area of the microscope slide, dried, heat-fixed by passing through a flame, and auramine stained by flooding with 0.1% auramine (15 minutes) and 0.5% acid-alcohol decolorizing solution (2 minutes); rinsing with distilled water; flooding with 0.5% potassium permanganate (2 minutes); rinsing with distilled water; drying; storing in the dark; and performing microscopy within 6 hours [25].

### Microscopy

Visible bacteria were counted in 100 consecutive microscopy fields at ×1000 magnification (approximately 2 mm^2^ [26]). Concentrations of stained bacteria per milliliter sample were calculated and are termed “viability” and “acid-fast microscopy.”

#### Cultures

Cultures used the microscopic-observation drug susceptibility (MODS) technique [27, 28] adapted to be quantitative [5]. A 1:10 dilution of 50 µL decontaminated sputum in 450 µL culture broth [28] was mixed by pipetting; 50 µL was mixed into a 1:100 dilution well, and then into a 1:1000 dilution well. Cultures were sealed in unsupplemented room air, incubated at 37°C, and examined with an inverted microscope for cording colonies to determine positivity. This is labor intensive and was performed 3 times weekly [5], making time to culture positivity data poorly discriminatory. Consequently, the primary quantification of *M. tuberculosis* growth was colony-forming units as described elsewhere [5], divided by the sample volume (mL) inoculated into that culture, and termed “quantitative culture.” Positive cultures were confirmed to be *M. tuberculosis* with the Capilia assay (Tauns, Tokyo, Japan).

### Drug Susceptibility Testing

Isoniazid and rifampicin susceptibility of pretreatment samples were analyzed with the standard nonquantitative MODS technique [27, 28] and the tetrazolium microplate assay (TEMA) [29].

### Analysis

Recruitment took place over 12 months, so recruitment and follow-up samples from different patients were analyzed concurrently. Staff were blinded to all other clinical and laboratory data. Because artifacts can cause weakly false-positive microscopy results, and in accordance with Peruvian policy for acid-fast microscopy, all microscopy was considered positive if ≥10 objects with the appearance of *M. tuberculosis* were seen [21]. The international MODS protocol determined that ≥2 colonies indicated a positive result because single colonies may result from cross-contamination [30]. Bacterial counts were exponentially distributed, so were transformed to their base-10 logarithm (after zero values were transformed to the midpoint between zero and the detection threshold) and are reported as geometric means with 95% confidence intervals (CIs). Parametric data were summarized as mean with standard deviation (SD). Nonparametric data were summarized as median and interquartile range. Linear regression used random effects to adjust for between-patient variations. The study sample size was resource limited, and treatment response power calculations were not done. All analyses were 2-tailed and performed with Stata software version 11. All data are reported to 2 significant figures.

## RESULTS

### Pretreatment Sputum

Pretreatment sputum was available from 35 patients whose characteristics are shown in Table 1. Our research protocol requirement for patients to collect sputum for 12 hours before commencing treatment slowed recruitment because tuberculosis treatment was usually started immediately following diagnosis. This logistical limitation was unrelated to patient characteristics, so participants were locally representative. All participants had positive culture and acid-fast microscopy results and had viability-positive bacteria visualized; 94% (33/35) had viability above the threshold for positivity.

### Sputum Samples

**Figure 1.**
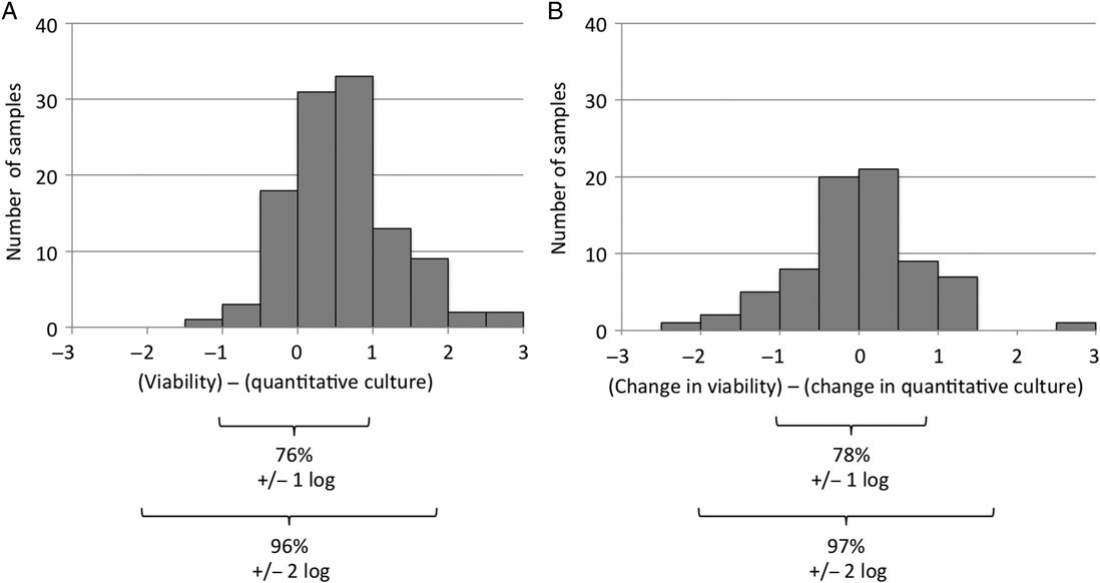
Tuberculosis quantitative viability microscopy prediction of quantitative culture results. Histograms show for each sputum sample the difference between quantitative tuberculosis viability microscopy results minus quantitative culture results (*A*) and the change in quantitative tuberculosis viability microscopy results minus the change in quantitative culture results (*B*) during each interval of 3 days of treatment (baseline 0 to 3 days, 3 to 6 days, and 6 to 9 days of follow-up). All results are shown on a log scale (“log” indicates base-10 logarithm); 76%–78% of results agreed within ±1 logarithm and 96%–97% agreed within ±2 logarithms. Microscopy results are geometric means of triplicate identical slides from each sample (see also Supplementary Figure 1).

All sputa underwent microscopy. Sputa were available for 89% (124/140) of the intended samples on days 0, 3, 6, and 9. Quantitative cultures provided interpretable results for 90% (112/124) of samples (Table 1). Viability predicted quantitative culture (Figure 1*A*), such that 76% of results agreed within ±1 logarithm and 96% within ±2 logarithms, and there was good correlation (*r*_S_ = 0.85; *P* < .001). Similarly, 78% of the changes in viability during each interval of 3 days’ treatment (baseline 0 to 3 days, 3 to 6 days, and 6 to 9 days of follow-up) predicted within ±1 logarithm of the changes in quantitative culture (Figure 1*B*). Viability correlated with time to culture positivity (*r*_S_ = −0.50; *P* < .0001), and time to culture positivity correlated with quantitative culture (*r*_S_ = −0.58; *P* < .0001).

### Drug Susceptibility Testing

DST results were 100% concordant between MODS and TEMA.

### Non-Multidrug-Resistant Tuberculosis

**Figure 2.**
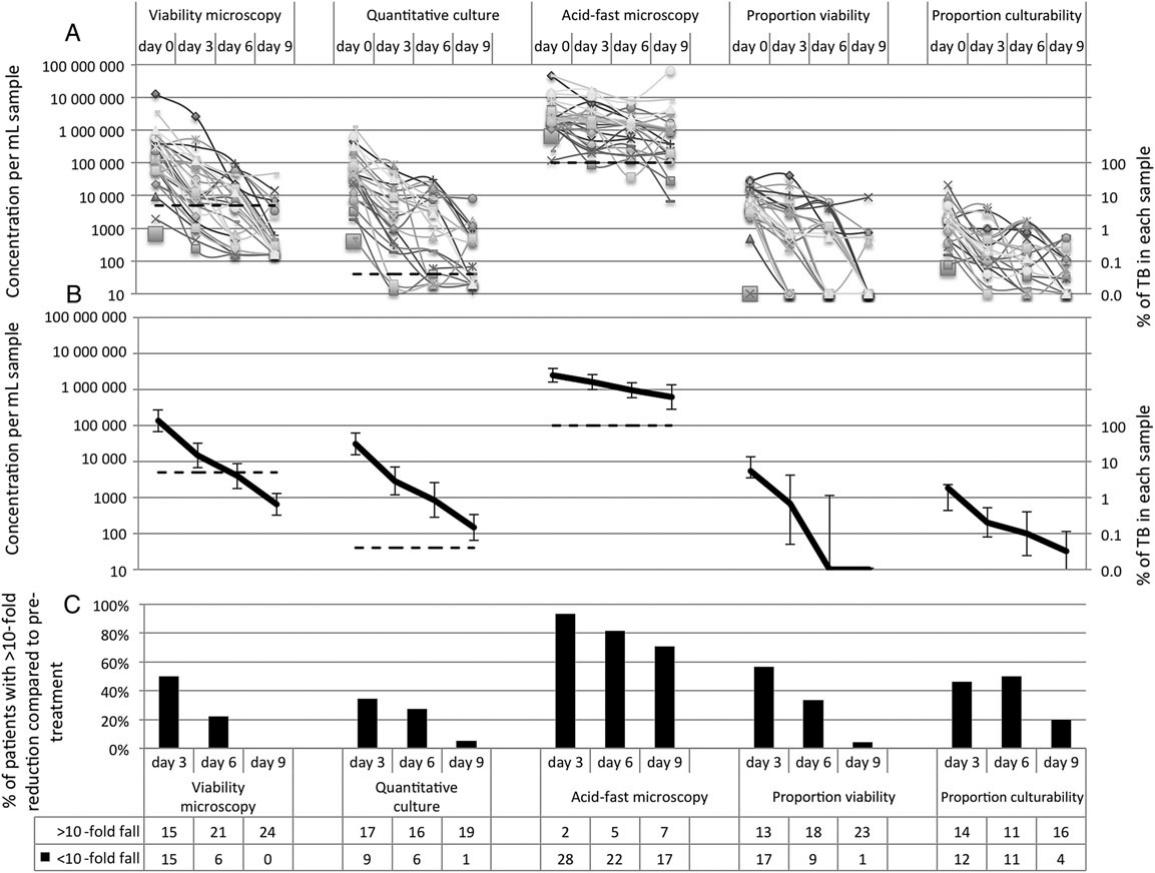
Treatment response for 31 patients with non-multidrug-resistant tuberculosis (TB) on days 0, 3, 6, and 9 of tuberculosis treatment for each patient (*A*), mean of all patients (*B*), and percentage of patients with <10-fold reduction (*C*). The x-axis shows days of treatment. Dashed lines indicate cutoffs for positivity. Proportion viability was calculated by dividing concentrations of viability-positive bacteria by concentrations of acid-fast microscopy–positive bacteria. Similarly, proportion culturability was calculated by dividing quantitative culture results by concentrations of acid-fast microscopy–positive bacteria. Microscopy results are geometric means of triplicate identical slides from each sample (see also Supplementary Figure 2).

Eighty-nine percent (31/35) of patients had non-multidrug-resistant (MDR) tuberculosis, including 2 patients with isoniazid monoresistance (Figure 2). Compared with pretreatment, mean viability for patients with non-MDR tuberculosis after 3, 6, and 9 days of treatment reduced 11-fold, 43-fold, and 250-fold, respectively (all *P* < .001). By treatment day 9, viability reduced >10-fold in all patients with non-MDR tuberculosis and became negative for 79% (19/24). Quantitative culture mean results for patients with non-MDR tuberculosis after 3, 6, and 9 days of treatment reduced 15-fold, 46-fold, and 190-fold, respectively, compared with pretreatment (all *P* < .001). By treatment day 9, quantitative culture had reduced >10-fold in 95% (19/20) of patients with non-MDR tuberculosis and became negative for 38% (8/21). Acid-fast microscopy mean results fell <5-fold throughout early treatment, so by treatment day 9, acid-fast microscopy had reduced >10-fold in only 25% (6/24) of patients with non-MDR tuberculosis and became negative in only 13% (3/24). Regression analysis (Table 2) demonstrated that in patients with non-MDR tuberculosis, viability approximately halved daily (0.27 log; *P* < .001), as did quantitative culture (0.27 log; *P* < .001). Acid-fast microscopy changed little, reducing by only 0.07 log daily (*P* = .04).

**Table 2.** Regression Analysis of Laboratory Results During Early Treatment

Variables	Viability Microscopy			Quantitative Culture			Acid-Fast Microscopy		
	Coefficient (Difference in Log Concentration)	95% CI	*P* Value	Coefficient (Difference in Log Concentration)	95% CI	*P* Value	Coefficient (Difference in Log Concentration)	95% CI	*P* Value
Day 0 (pretreatment): difference in concentration for MDR tuberculosis vs non-MDR tuberculosis	−0.48	−1.2 to .27	.2	−0.39	−1.3 to .55	.4	−0.073	−.62 to .48	.8
Non-MDR tuberculosis: daily change in concentration during treatment	−**0.27**	−**.29 to** −**.25**	**<.001**	−**0.27**	−**.29 to** −**.25**	**<.001**	−**0.070**	−**.085 to** −**.056**	**<.001**
MDR tuberculosis: daily change in concentration during treatment	−0.028	−.10 to .044	.4	−0.019	−.095 to .057	.6	−**0.056**	−**.11** to **.0033**	**.04**

This table demonstrates the effects of MDR tuberculosis and days of treatment on laboratory results. There was very strong evidence for an interaction between MDR tuberculosis and daily change in concentration for both viability microscopy and quantitative culture (both *P* < .001), but no evidence of an interaction for acid-fast microscopy (*P* = .6). Microscopy results are geometric means of triplicate identical slides from each sample (see also Supplementary Table 2). The values in bold are statistically significant.

Abbreviations: CI, confidence interval; log, base-10 logarithm; MDR, multidrug resistant.

### MDR Tuberculosis

**Figure 3.**
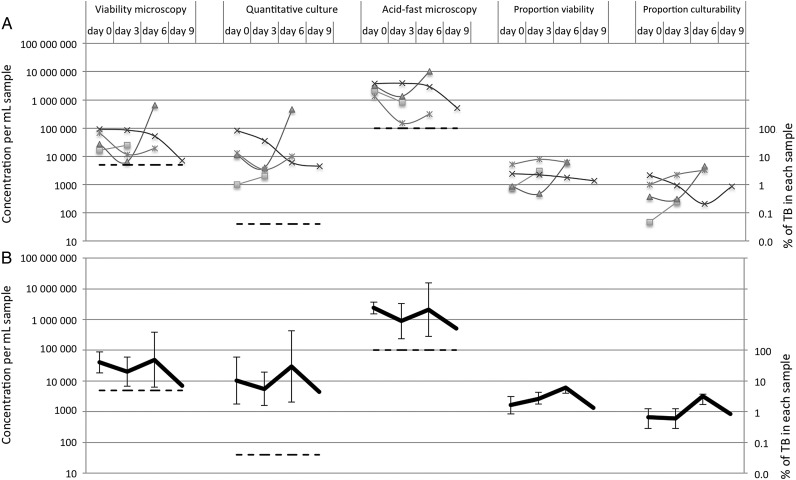
Treatment response for 4 patients with multidrug-resistant tuberculosis (TB) on days 0, 3, 6, and 9 of tuberculosis treatment for each patient (*A*) and mean of all patients (*B*). The x-axis shows days of treatment. Dashed lines indicate cutoffs for positivity. Proportion viability was calculated by dividing concentrations of viability-positive bacteria by concentrations of acid-fast microscopy–positive bacteria. Similarly, proportion culturability was calculated by dividing concentrations of culture-positive bacteria by concentrations of acid-fast microscopy–positive bacteria. Microscopy results are geometric means of triplicate identical slides from each sample (see also Supplementary Figure 3).

In the 11% (4/35) of patients with MDR tuberculosis, there were no significant changes in viability (*P* = .4) or quantitative culture (*P* = .6) during treatment (Table 2), but only 12 samples were available (Figure 3).

### Non-MDR Tuberculosis Versus MDR Tuberculosis

Changes in viability and quantitative culture during treatment differed significantly for patients with non-MDR tuberculosis vs MDR tuberculosis (both *P* < .001; Table 2). Viability changes were significantly greater for non-MDR tuberculosis than MDR tuberculosis after 3, 6, and 9 days of treatment (all *P* < .001). Quantitative culture changes were significantly greater for non-MDR tuberculosis vs MDR tuberculosis after 6 and 9 days treatment (*P* = .001 and *P* = .04, respectively). Acid-fast microscopy was similar comparing patients with non-MDR tuberculosis vs MDR tuberculosis overall and after 3, 6, and 9 days treatment (*P* = .6, *P* = .2, *P* = .08, and *P* = .3, respectively).

### Proportion Viability and Culturability

For every decontaminated sputum, per-milliliter concentrations were greater for acid-fast microscopy than for viability and quantitative culture (both *P* < .001). Proportion viability was calculated by dividing concentrations of viability-positive bacteria by concentrations of acid-fast microscopy–positive bacteria. Similarly, proportion culturability was calculated by dividing the quantitative culture results by concentrations of acid-fast microscopy–positive bacteria. For patients with non-MDR tuberculosis (Figure 2), proportion viability and proportion culturability fell during treatment (both *P* < .001). Consequently, for patients with non-MDR tuberculosis by treatment day 9, there had been a >10-fold fall in proportion viability for 96% (23/24) of patients and proportion culturability for 80% (16/20) of patients. In contrast, for the few patients with MDR tuberculosis (Figure 3), there was no significant change in proportion viability or proportion culturability during treatment (both *P* > .4).

### Microscopy Reproducibility

**Figure 4.**
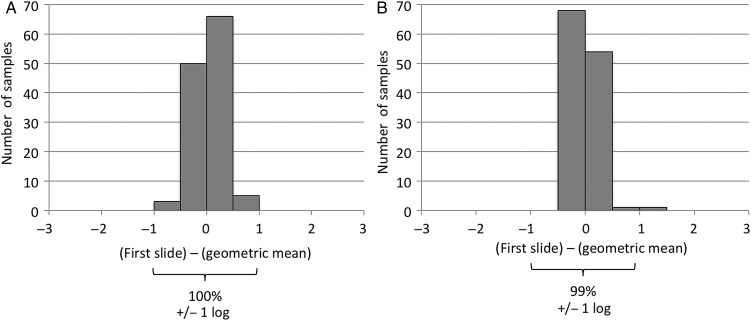
Comparison of results for first microscopy slides vs geometric means. Histograms showing for each sputum sample the difference between quantitative tuberculosis viability microscopy results (*A*) and the acid-fast microscopy results for first microscopy slides vs geometric means of the results (*B*) for the first, second and third slides. All results are shown on a log scale (“log” indicates base-10 logarithm); 100% of results agreed within ±1 logarithm.

Linear regression demonstrated that variation between triplicate slides accounted for no more than 5% of the variation between microscopy results (ρ = 0.95). Consequently, 100% of first slides predicted geometric mean results of triplicate (ie, first, second, and third) slides within ±1 logarithm for viability (Figure 4*A*) and 99% for acid-fast microscopy (Figure 4*B*). The results of all the above-mentioned analyses were little changed by using only first slide results (see Supplementary Data).

### Clinical Outcomes

All patients had outcome assessed at the time of treatment completion. All 31 patients with non-MDR tuberculosis were cured; all but 1 were followed up for ≥3 years, and no recurrences were identified. All 4 patients with MDR tuberculosis had adverse outcomes: 1 patient had treatment suspended and the 3 other patients died. These deaths occurred 22 days, 10 months, and 11 months after initially commencing therapy.

## DISCUSSION

Tuberculosis quantitative viability microscopy with fluorescein diacetate staining for metabolically active bacteria accurately predicted the concentration of culturable *M. tuberculosis* in sputum. Viability microscopy took <1 hour and was less resource-intensive, requiring only basic skills that were already available, whereas quantitative culture took weeks and required more laboratory equipment and expertise. Over the first 9 days of tuberculosis treatment, viability microscopy reliably predicted changes in culturable *M. tuberculosis* concentrations.

Viability and quantitative culture changes appeared to separate into 2 distinct patterns during early treatment depending on whether patients had MDR tuberculosis. For patients with non-MDR tuberculosis, after 3 days, treatment mean viability and quantitative culture results fell by >90% and by day 9 by 99.6%. This concurs with early bactericidal activity and serial sputum colony count studies of tuberculosis treatment response, which reported exponential isoniazid-mediated decline in culturable *M. tuberculosis* during the first days of treatment, followed by slower rifampicin-mediated killing [1, 3, 31, 32]. Acid-fast microscopy changed little during early treatment, so results diverged from viability and quantitative culture as treatment continued [2]. In contrast to these results for patients with non-MDR tuberculosis, viability and quantitative culture for the few patients with MDR tuberculosis differed significantly because they did not decrease during early treatment.

Studies in Bangladesh and Thailand reported that “positive-vs-negative” viability microscopy after 2 months treatment identified patients needing DST for probable MDR tuberculosis [33–35]. Here we demonstrate the value of a modified quantitative viability microscopy technique to screen patients with tuberculosis for poor treatment response and MDR tuberculosis within 9 days of starting treatment. Patients with MDR tuberculosis have worse outcomes, and viability microscopy during early treatment may allow the sparse capacity for DST to be provided to the patients most likely to have MDR tuberculosis [4]. This has the potential to improve patient outcomes and reduce MDR tuberculosis dissemination [36]. Future research should also assess whether viability microscopy during early treatment identifies treatment that is failing because of factors such as malabsorption or inadequate adherence, which are difficult to detect.

We analyzed viability microscopy not only as viable *M. tuberculosis* concentrations, but also as proportion viability that was calculated as the viability divided by acid-fast microscopy results. We included this approach in case low-quality sputum specimens (or saliva submitted instead of sputum) caused misleading results, but this did not occur in our study, which used sputum samples collected at home over 12-hour periods. Our results suggest that the simpler assessment of the concentration of viable *M. tuberculosis* was as reliable, more consistent between samples, and easier to analyze than proportion viability. To reduce the potential effect of random variation between microscopy slides, we empirically performed all microscopy tests on identical triplicate slides from each specimen. However, this increased workload, and we found that results from only the first microscopy slide gave similar results, perhaps because sputum homogenization and processing were carefully standardized to reduce slide-to-slide variations and the risk of bias comparing results between different techniques. We therefore recommend that in the future only a single slide is prepared for each microscopy technique from each sample.

The skills required and reagent costs for viability microscopy were similar to those in routine use in many settings for acid-fast microscopy. A fluorescent microscope is required but their availability is increasing and costs falling because of LED (light-emitting diode) technology and because the World Health Organization has recommended they be used to increase the sensitivity of acid-fast microscopy for routine tuberculosis diagnostic testing [37]. Although we used a 37**°**C incubator to reduce potential day-to-day variations in this research, inexpensive portable incubators may be fabricated or purchased, and we are evaluating whether incubation may be omitted. Thus, viability microscopy may be considered an appropriate-technology test for use in basic laboratories in resource-constrained settings.

Study limitations include that microscopy was only used in patients with initially acid-fast microscopy–positive tuberculosis, which corresponded with >100 000 *M. tuberculosis*/mL sputum (Figures 2 and 3), as in previous research [26]. However, filters or centrifugation concentrate *M. tuberculosis* from sputum and may reduce this limitation [38]. Other limitations included the study size because of numbers of patients recruited, missing samples when patients could not be located, the lack of pyrazinamide susceptibility testing, and some uninterpretable contaminated cultures. The culture contamination rate was within the range of previous studies using MODS but was higher than average [39], perhaps because samples were collected over a longer-than-typical interval (12 hours). The small number of patients with MDR tuberculosis and their few samples are important limitations to be addressed in future research. Despite these limitations, the study size was sufficient to demonstrate statistically significant viability differences between patients with non-MDR tuberculosis vs MDR tuberculosis. To extend these findings, we are commencing a larger multisite study assessing the potential programmatic value of these findings by including more patients with MDR tuberculosis and human immunodeficiency virus coinfection and assessing their single “spot” (not 12-hour) sputum tested with single-slide viability microscopy prior to and 14 days after commencing treatment.

Here we show that viability microscopy rapidly demonstrated when treatment killed *M. tuberculosis* in patients' sputum, rendering that sputum culture negative and presumably noninfectious. It is frequently difficult to assess when the infectiousness of patients is being reduced by treatment. Consequently, infection control precautions may either be prolonged until sputum acid-fast or culture results become negative, or stopped based on unreliable indicators such as symptomatic response or an arbitrary number of days of treatment [40]. Assessing patient infectiousness is challenging [36], and in separate research we are assessing whether the wide, naturally occurring variations in *M. tuberculosis* proportion viability in pretreatment sputum had implications for patients' infectiousness to their contacts. However, tuberculosis culture is the gold-standard test for the infectiousness of sputum, and infection control assessments are often based upon sputum culture results. Thus, by rapidly predicting quantitative culture results, viability microscopy may provide timely evidence on which to base infection control decisions.

Tuberculosis quantitative viability microscopy predicted in 1 hour the results of sputum culture that became available weeks later. In the resource-constrained settings where most tuberculosis occurs, this appropriate-technology technique had promising results for informing decisions concerning DST and changes in treatment and infection control measures.

## Supplementary Data

Supplementary materials are available at *Clinical Infectious Diseases* online (http://cid.oxfordjournals.org). Supplementary materials consist of data provided by the author that are published to benefit the reader. The posted materials are not copyedited. The contents of all supplementary data are the sole responsibility of the authors. Questions or messages regarding errors should be addressed to the author.

Supplementary Data

## References

[1] Jindani A Doré CJ Mitchison DA Bactericidal and sterilizing activities of antituberculosis drugs during the first 14 days. Am J Respir Crit Care Med 2003; 167:1348–54. 12519740 10.1164/rccm.200210-1125OC

[2] Hobby GL Holman AP Iseman MD Jones JM Enumeration of tubercle bacilli in sputum of patients with pulmonary tuberculosis. Antimicrob Agents Chemother 1973; 4:94–104. 4208508 10.1128/aac.4.2.94 PMC444512

[3] Davies GR Brindle R Khoo SH Aarons LJ Use of nonlinear mixed-effects analysis for improved precision of early pharmacodynamic measures in tuberculosis treatment. Antimicrob Agents Chemother 2006; 50:3154–6. 16940116 10.1128/AAC.00774-05 PMC1563554

[4] World Health Organization. Global tuberculosis report 2013.

[5] Grandjean L Martin L Gilman RH Tuberculosis diagnosis and multidrug resistance testing by direct sputum culture in selective broth without decontamination or centrifugation. J Clin Microbiol 2008; 46:2339–44. 18448689 10.1128/JCM.02476-07 PMC2446921

[6] Evans CA GeneXpert—a game-changer for tuberculosis control? PLoS Med 2011; 8:e1001064. 21814497 10.1371/journal.pmed.1001064 PMC3144196

[7] Friedrich SO Rachow A Saathoff E Assessment of the sensitivity and specificity of Xpert MTB/RIF assay as an early sputum biomarker of response to tuberculosis treatment. Lancet Respir Med 2013; 1:462–70. 24429244 10.1016/S2213-2600(13)70119-X

[8] Desjardin LE Perkins MD Wolski K Measurement of sputum *Mycobacterium tuberculosis* messenger RNA as a surrogate for response to chemotherapy. Am J Respir Crit Care Med 1999; 160:203–10. 10390401 10.1164/ajrccm.160.1.9811006

[9] Escombe AR Oeser C Gilman RH The detection of airborne transmission of tuberculosis from HIV-infected patients, using an in vivo air sampling model. Clin Infect Dis 2010; 44:1349–57. 17443474 10.1086/515397 PMC2912511

[10] Kvach J Veras J A fluorescent staining procedure for determining the viability of mycobacterial cells. Int J Lepr 1982; 50:183. 6180992

[11] Katoch V Katoch K Ramanathan U Effect of chemotherapy on viability of *Mycobacterium leprae* as determined by ATP content, morphological index and FDA-EB flourescent staining. Int J Lepr 1989; 57:615–21. 2476522

[12] Harada S Numata N Application of FDA/EB staining for the detection of viable or non-viable mycobacteria in clinical specimens. Kekkaku 1992; 67:113–7. 1372666

[13] Kinomoto M Development of slide-method to distinguish alive and dead mycobacteria by flourescent staining. Kekkaku 1999; 74:599–609. 10487028

[14] Kirk SM Schell RF Moore AV Callister SM Mazurek GH Flow cytometric testing of susceptibilities of *Mycobacterium tuberculosis* isolates to ethambutol, isoniazid, and rifampin in 24 hours. J Clin Microbiol 1998; 36:1568–73. 9620378 10.1128/jcm.36.6.1568-1573.1998 PMC104878

[15] Moore AV Kirk SM Callister SM Mazurek H Schell RF Mazurek GH Safe determination of susceptibility of *Mycobacterium tuberculosis* to antimycobacterial agents by flow cytometry. J Clin Microbiol 1999; 37:479–83. 9986799 10.1128/jcm.37.3.479-483.1999 PMC84439

[16] Norden MA Kurzynski TA Bownds SE Callister SM Schell RF Rapid susceptibility testing of *Mycobacterium tuberculosis* (H37Ra) by flow cytometry. J Clin Microbiol 1995; 33:1231–7. 7615733 10.1128/jcm.33.5.1231-1237.1995 PMC228136

[17] Bravard M Sherman JM Martin L Sputum vital stain microscopy to predict culture results and infectiousness. In: Proceedings of the 41st world annual Union Against Tuberculosis and Lung Diseases conference, Berlin, Germany, 2010; 13:S85 Available at: http://www.ifhad.org/publications/. Accessed 31 December 2014.

[18] Sherman JM Montoya R Gilman RH Rapid monitoring of anti-tuberculosis therapy using fluorescein diacetate microscopy a simple method to determine infectiousness andscreen for drug resistance. In: Proceedings of Royal Society of Tropical Medicine and Hygiene centennial conference; London, UK, 2007.

[19] Sherman JM Montoya R Gilman RH Montoring anti-tuberculosis therapy with fluorescein diacetate (FDA) microscopy rapidly determines infectiousness and screen for drug resistance. In: Proceedings of Medecins Sans Frontieres (MSF); Campaign for Access to Essential Medicines. Symposium on TB FIELD Diagnostics: “Dying for a test;” Cape Town, South Africa, 2007 Available at: http://www.ifhad.org/publications/. Accessed 31 December 2014.

[20] Kawai V Soto G Gilman RH Tuberculosis mortality, drug resistance, and infectiousness in patents with and without HIV infection in Peru. Am J Trop Med Hyg 2010; 75:1027–33. 17172361 PMC2912515

[21] Ministerio de salud, Peru. Norma tecnica de salud para el control de la tuberculosis. Lima: Ministry of Health, 2006.

[22] Rocha C Montoya R Zevallos K The Innovative Socio-economic Interventions Against Tuberculosis (ISIAT) project: an operational assessment. Int J Tuberc Lung Dis 2011; 15(suppl 2):S50–7. 21740659 10.5588/ijtld.10.0447 PMC3160483

[23] Sherman JM Tovar M Gilman RH Using treatment failure to screen for MDRTB is associated with TB recurrence, death and transmission. In: Proceedings of 55th annual conference of the American Society of Tropical Medicine and Hygiene, Atlanta, GA, 2006: S312.

[24] Datiko DG Lindtjørn B Tuberculosis recurrence in smear-positive patients cured under DOTS in southern Ethiopia: retrospective cohort study. BMC Public Health 2009; 9:348. 19765291 10.1186/1471-2458-9-348 PMC2754462

[25] Kent PT Public health mycobacteriology: a guide for the level III laboratory. Atlanta, GA: US Department of Health and Human Services, Public Health Service, Centers for Disease Control, 1985 Available at: https://catalyst.library.jhu.edu/catalog/bib_1853570 Accessed 12 February 2014.

[26] Toman K Toman's tuberculosis; case detection, treatment and monitoring—questions and answers. 2nd ed. Geneva, Switzerland: WHO, 2004.

[27] Moore DAJ Evans CAW Gilman RH Microscopic-observation drug-susceptibility assay for the diagnosis of TB. N Engl J Med 2006; 355:1539–50. 17035648 10.1056/NEJMoa055524 PMC1780278

[28] Caviedes L Lee TS Gilman RH Rapid, efficient detection and drug susceptibility testing of *Mycobacterium tuberculosis* in sputum by microscopic observation of broth cultures. The Tuberculosis Working Group in Peru. J Clin Microbiol 2000; 38:1203–8. 10699023 10.1128/jcm.38.3.1203-1208.2000 PMC86377

[29] Caviedes L Delgado J Gilman RH Tetrazolium microplate assay as a rapid and inexpensive colorimetric method for determination of antibiotic susceptibility of *Mycobacterium tuberculosis*. J Clin Microbiol 2002; 40:1873–4. 11980982 10.1128/JCM.40.5.1873-1874.2002 PMC130930

[30] Moore DAJ Caviedes L Gilman RH Infrequent MODS TB culture cross-contamination in a high-burden resource-poor setting. Diagn Microbiol Infect Dis 2006; 56:35–43. 16678991 10.1016/j.diagmicrobio.2006.03.009 PMC2912514

[31] Chigutsa E Patel K Denti P A time-to-event pharmacodynamic model describing treatment response in patients with pulmonary tuberculosis using days to positivity in automated liquid mycobacterial culture. Antimicrob Agents Chemother 2013; 57:789. 23183433 10.1128/AAC.01876-12 PMC3553722

[32] Bark CM Gitta P Johnson JL Differences in EBA in the first two days of standard anti-tuberculosis treatment in different geographic regions. Int J Tuberc Lung Dis 2003:1006–7. 10.5588/ijtld.13.0826 PMC4415604 25199023

[33] Schramm B Hewison C Bonte L Field evaluation of a simple fluorescence method for detection of viable *Mycobacterium tuberculosis* in sputum specimens during treatment follow-up. J Clin Microbiol 2012; 50:2788–90. 22649015 10.1128/JCM.01232-12 PMC3421534

[34] Salim AH Aung KJM Hossain MA Van Deun A Early and rapid microscopy-based diagnosis of true treatment failure and MDR-TB. Int J Tuberc Lung Dis 2006; 10:1248–54. 17131784

[35] Van Deun A Maug AKJ Hossain A Gumusboga M De Jong BC Fluorescein diacetate vital staining allows earlier diagnosis of rifampicin-resistant tuberculosis. Int J Tuberc Lung Dis 2012; 16:1174–9. 22747903 10.5588/ijtld.11.0166

[36] Escombe AR Moore DAJ Gilman RH The infectiousness of tuberculosis patients coinfected with HIV. PLoS Med 2008; 5:e188. 18798687 10.1371/journal.pmed.0050188 PMC2535657

[37] World Health Organization. Fluorescent light-emitting diode (LED) microscopy for diagnosis of tuberculosis policy, 2011 Available at: http://www.who.int/tb/publications/2011/led_microscopy_diagnosis_9789241501613/en/ Accessed 20 February 2014. 23586120

[38] Ramos E Schumacher SG Siedner M Optimizing tuberculosis testing for basic laboratories. Am J Trop Med Hyg 2010; 83:896–901. 20889887 10.4269/ajtmh.2010.09-0566 PMC2946764

[39] Leung E Minion J Benedetti A Pai M Menzies D Microcolony culture techniques for tuberculosis diagnosis : a systematic review. Int J Tuberc Lung Dis 2011; 16:16–23. 21986554 10.5588/ijtld.10.0065

[40] World Health Organization; Granich R Binkin N Jarvis W Simone P Rieder H Guideline for the prevention of tuberculosis. Geneva, Switzerland: 1999: 31 Available at: http://www.who.int/tb/publications/who_tb_99_269.pdf?ua=1 Accessed 19 May 2014.

