# Supplementary material for: Clinical Evaluation of Tuberculosis Viability Microscopy for Assessing Treatment Response
Source: Clin Infect Dis. 2014 Dec 23;60(8):1186–95. doi: 10.1093/cid/ciu1153 (PMC4370166; doi:10.1093/cid/ciu1153)
Supplement: Supplementary Data [file supp_60_8_1186__index.html]

Clinical Evaluation of Tuberculosis Viability Microscopy for Assessing Treatment Response — Clinical Evaluation of Tuberculosis Viability Microscopy for Assessing Treatment Response — Supplementary Data 

# Clinical Evaluation of Tuberculosis Viability Microscopy for Assessing Treatment Response

## Supplementary Data

Supplementary Data

**Files in this Data Supplement:**

- Supplementary Data - Docx file
- Supplementary Figure 1 - tif file
- Supplementary Figure 2 - tif file
- Supplementary Figure 3 - tif file
