# Supplementary material for: Clinical Evaluation of Tuberculosis Viability Microscopy for Assessing Treatment Response
Source: Clin Infect Dis. 2014 Dec 23;60(8):1186–95. doi: 10.1093/cid/ciu1153 (PMC4370166; doi:10.1093/cid/ciu1153)
Supplement: Supplementary Data [file supp_ciu1153_ciu1153supp.docx]

**Online Table 1.** The treatment provided to patients in this study by the Peruvian national TB control program is detailed in the TB program guide [21]. In summary, this constituted:

|  | SCHEME 1 | | | |
| --- | --- | --- | --- | --- |
|  | INITIATION PHASE | | CONTINUATION PHASE | |
|  | 2 months of treatment 6 days per week | | 4 months of twice weekly treatment | |
|  | <50 kg | >50 kg | <50 kg | >50 kg |
|  | Dose (mg/kg) | Dose (mg) | Dose (mg/kg) | Dose (mg) |
| Rifampicin | 10 | 600 | 10 | 600 |
| Isoniazid | 5 | 300 | 15 | 800 |
| Ethambutol | 20 | 1200 | - | - |
| Pyrazinamide | 25 | 1500 | - | - |
|  |  |  |  |  |
|  |  |  |  |  |
|  | SCHEME 2  Patients who have had previous TB treatment for more than 30 days | | | |
|  | INITIATION PHASE | | CONTINUATION PHASE | |
|  | 3 months of treatment 6 days per week | | 5 months of twice weekly treatment | |
|  | <50 kg | >50kg | <50 kg | >50 kg |
|  | Dose (mg/kg) | Dose (mg) | Dose (mg/kg) | Dose (mg) |
| Rifampicin | 10 | 600 | 10 | 600 |
| Isoniazid | 5 | 300 | 15 | 800 |
| Ethambutol | 20 | 1200 | 20 | 2400 |
| Pyrazinamide | 25 | 1500 | - | - |
| Streptomycin* | 15 | 1000^+^ | - | - |

***** Streptomycin is given for the first 2 months of Scheme 2 initiation phase.

^+^ Patients aged over 60 years were given 750 mg.

**Online Table 2. Regression analysis of first slide laboratory results during early treatment.** This demonstrates the effects of multi-drug resistant tuberculosis (MDR-TB) and days of treatment on laboratory results. Note CI=confidence interval; log=base-10 logarithm. Microscopy results are for only the first slide from each sputum sample (see Table 2).

|  | Viability microscopy (first slide) | | | Quantitative Culture | | | Acid-fast microscopy (first slide) | | |
| --- | --- | --- | --- | --- | --- | --- | --- | --- | --- |
|  | Coefficient | 95%CI | p value | Coefficient | 95%CI | p value | Coefficient | 95%CI | p value |
|  | (difference in log |  |  | (difference in log |  |  | (difference in log |  |  |
|  | concentration) |  |  | concentration) |  |  | concentration) |  |  |
| (i) Day 0 (pre-treatment):  difference in concentration for  MDR-TB versus non-MDR-TB | -0.45 | -1.3 | 0.3 | -0.40 | -1.4 | 0.4 | -0.16 | -0.81 | 0.6 |
|  |  | to |  |  | to |  |  | to |  |
|  |  | 0.39 |  |  | 0.62 |  |  | 0.49 |  |
| (ii) Non-MDR-TB:  daily change in concentration during treatment | **-0.26** | **-0.29** | **<0.001** | **-0.27** | **-0.31** | **<0.001** | **-0.072** | **-0.10** | **<0.001** |
|  |  | **to** |  |  | **to** |  |  | **to** |  |
|  |  | **-0.22** |  |  | **-0.22** |  |  | **-0.043** |  |
| (iii) MDR-TB:  daily change in concentration during treatment | -0.044 | -0.17 | 0.5 | -0.0010 | -0.16 | 0.9 | -0.034 | -0.14 | 0.5 |
|  |  | to |  |  | to |  |  | to |  |
|  |  | 0.086 |  |  | 0.14 |  |  | 0.074 |  |

Note: there was very strong evidence for an interaction between MDR-TB and daily change in concentration for both viability microscopy and quantitative culture (p=0.002 and p=0.001 respectively), but no evidence of an interaction for acid-fast microscopy (p=0.5).

**Online Figure 1. TB quantitative viability microscopy prediction of quantitative culture results.** Histograms showing for each sputum sample the difference between (A) quantitative TB viability microscopy results minus quantitative culture results; and (B) the change in quantitative TB viability microscopy results minus the change in quantitative culture results during each interval of 3 days of treatment (baseline 0 days to 3 days, 3 to 6 days, and 6 to 9 days follow-up). All results are shown on a base-10 logarithm scale and ‘log’ indicates base-10 logarithm. 68% to 79% of results agreed within ±1 logarithm and, 95% to 96% agreed within ±2 logarithms. Microscopy results are for only the first slide from each sputum sample (see Figure 1).

**Online Figure 2. Treatment response for 31 patients with non-MDR-TB** on days 0, 3, 6 and 9 of TB treatment for: (a) each patient; (b) all patients; and (c) % of patients with <10-fold reduction. The horizontal-axis shows days of treatment. Dashed lines indicate cut-offs for positivity. Proportion-viability was calculated by dividing concentrations of viability-positive bacteria by concentrations of acid-fast microscopy-positive bacteria. Similarly, proportion-culturability was calculated by dividing concentrations of culture-positive bacteria by concentrations of acid-fast microscopy-positive bacteria. Microscopy results are for only the first slide from each sputum sample (see Figure 2).

**Online Figure 3. Treatment response for 4 patients with MDR-TB** on days 0, 3, 6 and 9 of TB treatment for: (a) each patient; and (b) all patients. The horizontal-axis shows days of treatment. Dashed lines indicate cut-offs for positivity. Proportion-viability was calculated by dividing concentrations of viability-positive bacteria by concentrations of acid-fast microscopy-positive bacteria. Similarly, proportion-culturability was calculated by dividing concentrations of culture-positive bacteria by concentrations of acid-fast microscopy-positive bacteria. Microscopy results are for only the first slide from each sputum sample (see Figure 3).
