# Supplementary figures and images for: Clinical Evaluation of Tuberculosis Viability Microscopy for Assessing Treatment Response
Source: Clin Infect Dis. 2014 Dec 23;60(8):1186–95. doi: 10.1093/cid/ciu1153 (PMC4370166; doi:10.1093/cid/ciu1153)

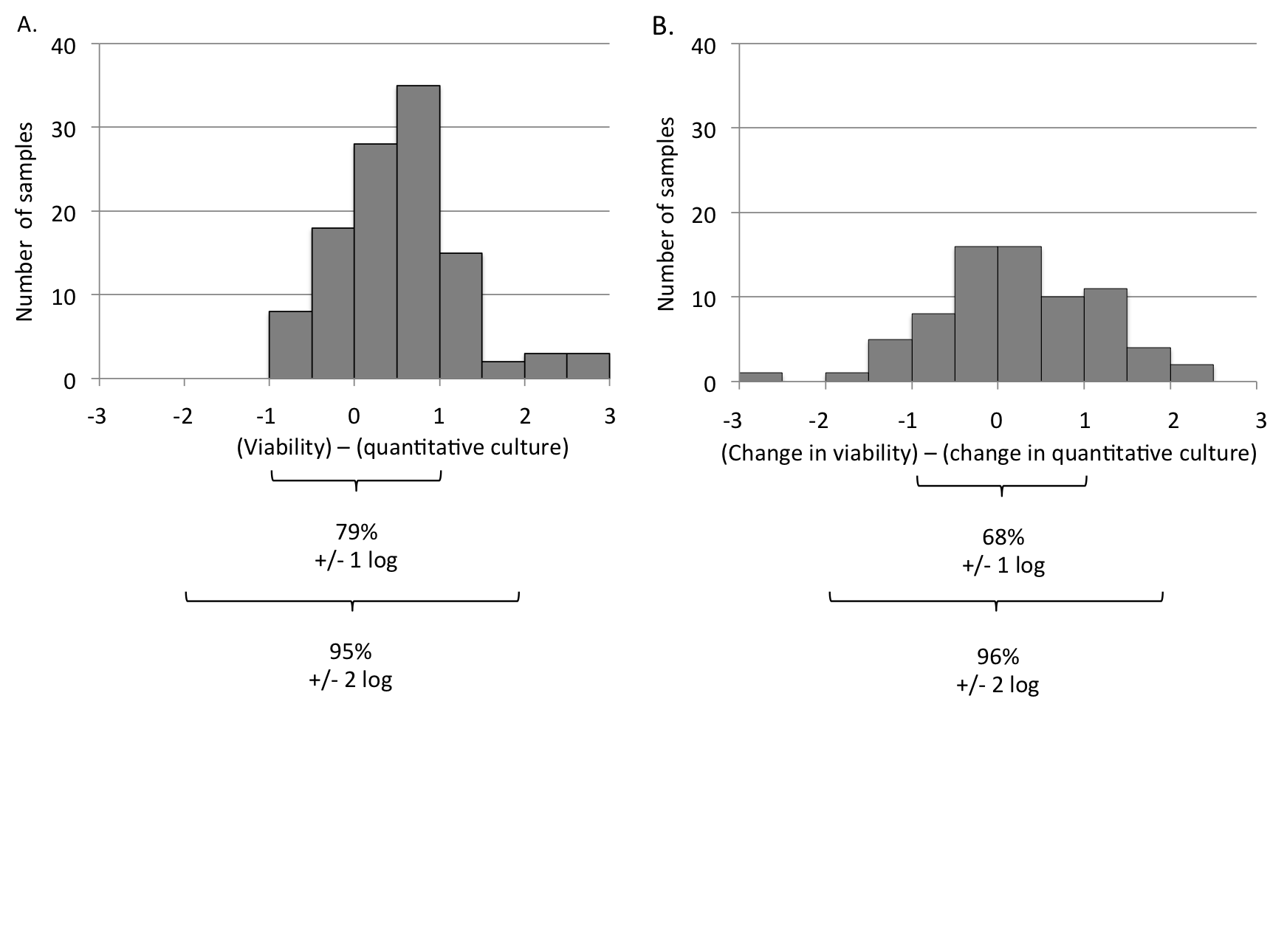

Supplement: Supplementary Data [file supp_ciu1153_ciu1153supp_fig1.tif]

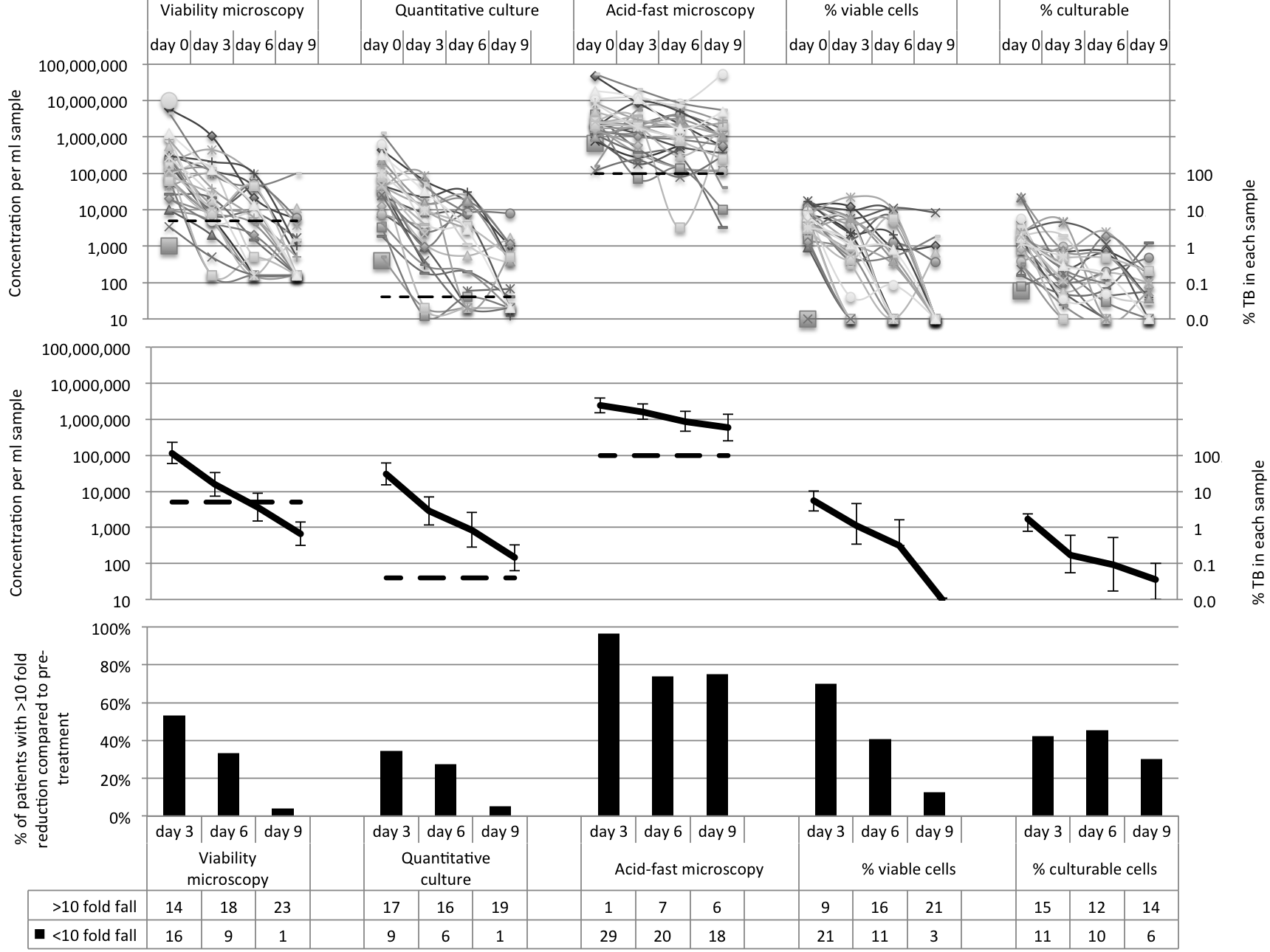

Supplement: Supplementary Data [file supp_ciu1153_ciu1153supp_fig2.tif]

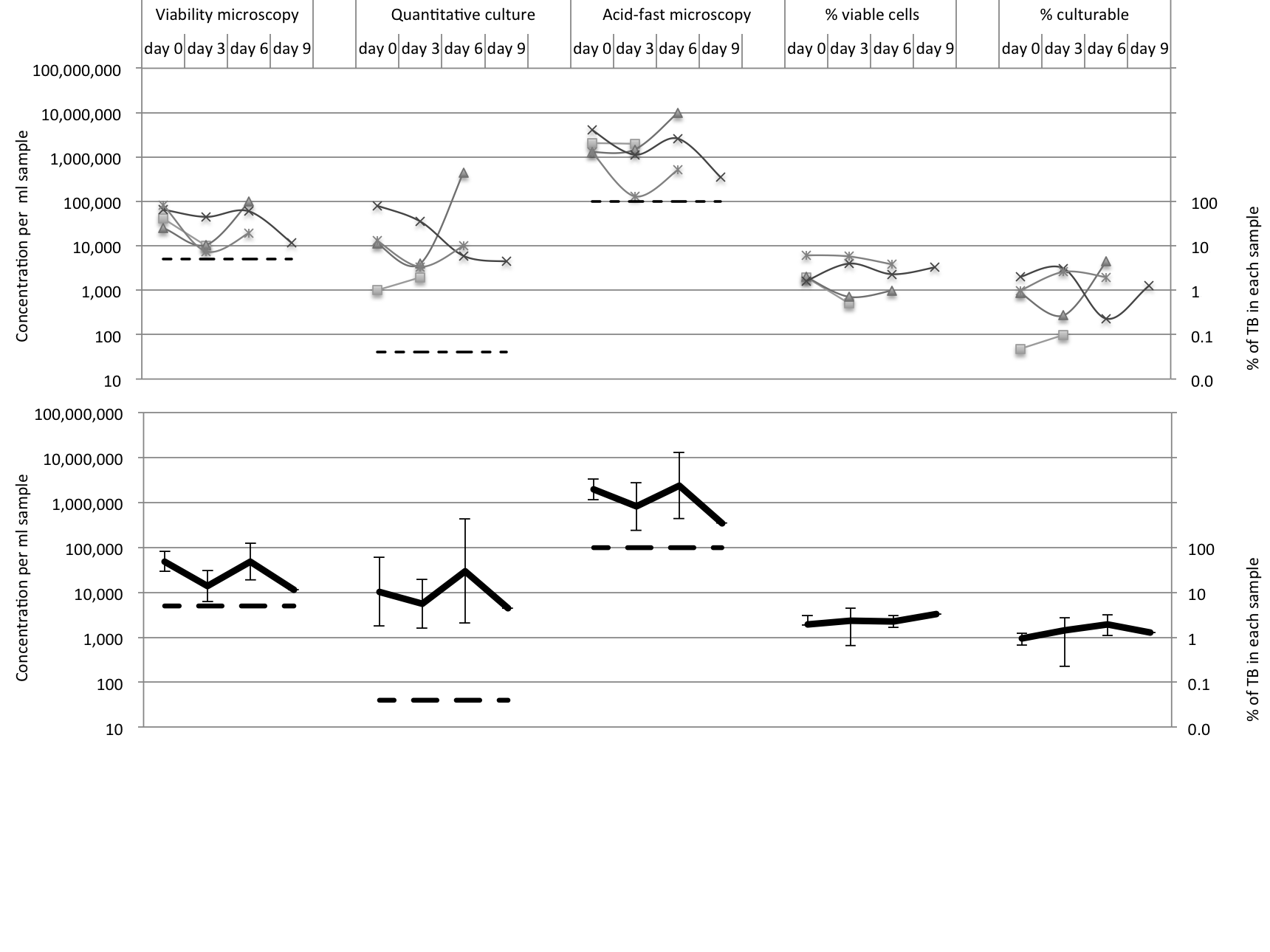

Supplement: Supplementary Data [file supp_ciu1153_ciu1153supp_fig3.tif]
